# Possible role of death receptor-mediated apoptosis by the E3 ubiquitin ligases Siah2 and POSH

**DOI:** 10.1186/1476-4598-10-57

**Published:** 2011-05-17

**Authors:** Perry A Christian, Michael V Fiandalo, Steven R Schwarze

**Affiliations:** 1 Markey Cancer Center and the Department of Molecular and Cellular Biochemistry, University of Kentucky, Lexington, KY 40536, USA

## Abstract

**Background:**

A functioning ubiquitin proteasome system (UPS) is essential for a number of diverse cellular processes and maintenance of overall cellular homeostasis. The ability of proteasome inhibitors, such as Velcade, to promote extrinsic apoptotic effects illustrates the importance of the ubiquitin proteasome system in the regulation of death receptor signaling. Here, we set out to define the UPS machinery, particularly the E3 ubiquitin ligases, that repress apoptosis through the extrinsic pathway. A cell-based genome-wide E3 ligase siRNA screen was established to monitor caspase-8 activity following the addition of TRAIL.

**Results:**

Data from the high-throughput screen revealed that targeting the RING-finger containing E3 ligase Siah2 as well as the signaling platform molecule POSH (SH3RF1) conferred robust caspase-8 activation in response to TRAIL stimulus. Silencing Siah2 or POSH in prostate cancer cells led to increased caspase activity and apoptosis in response to both TRAIL and Fas ligand. The E3 activity of Siah2 was responsible for mediating apoptosis resistance; while POSH protein levels were critical for maintaining viability. Further characterization of Siah2 revealed it to function downstream of early death receptor events in the apoptotic pathway. The observed apoptosis resistance provides one biological explanation for the induction of Siah2 and POSH reported in lung and prostate cancer, respectively. Expanding on an initial yeast-two-hybrid screen we have confirmed a physical interaction between E3 ligases Siah2 and POSH. Utilizing a yeast-two-hybrid mapping approach we have defined the spacer region of POSH, more specifically the RPxAxVxP motif encompassing amino acids 601-607, to be the site of Siah2 binding.

**Conclusions:**

The data presented here define POSH and Siah2 as important mediators of death receptor mediated apoptosis and suggest targeting the interaction of these two E3 ligases is a promising novel cancer therapeutic strategy.

## Background

Tight regulation of apoptosis is essential for maintaining tissue homeostasis. Altering this balance in favor of apoptosis resistance is a common feature in cancer cells [1], and overcoming cell death barriers is a primary goal of many chemotherapeutic treatments used in the clinic today. One proven strategy to induce apoptosis is through blocking components of the ubiquitin proteasome system (UPS).

The ubiquitin proteasome system is the major cellular pathway for regulated protein turnover. Ubiquitination and protein degradation allows the cell to respond quickly to extra- or intracellular signals, thereby maintaining cellular homeostasis. The addition of multiple ubiquitin moieties to a targeted protein "tags" the protein for degradation by the 26S proteasome. The mechanism by which multiple ubiquitin molecules are added to targeted substrates can be depicted as a three-step process. First, the ubiquitin activating enzyme (E1) catalyzes the formation of a C-terminal thiol ester in an ATP dependent reaction. The ubiquitin is then transferred to an E2 ubiquitin-conjugating enzyme via the formation of a thiol ester bond. Finally, the ubiquitin ligase enzymes (E3) catalyze the transfer of ubiquitin from the E2 to a lysine residue on the targeted protein via an isopeptide linkage. A polyubiquitinated protein is then recognized by the 19S regulatory subunit of the 26S proteasome and degraded by the 20S core particle.

Several strategies to block UPS components in the treatment of cancer have been devised. The initial approach was the development of the 26S proteasome inhibitor Velcade (bortezomib), which has been shown to induce apoptosis and is approved to treat multiple myeloma [2]. Subsequently, investigators have sought to promote the stability of numerous apoptosis-associated proteins, such as the p53 interaction with the E3 ligase Mdm2 [3]. Preventing proteasomal degradation of active caspases is another area that has shown promise. Polyubiquitination and subsequent degradation of active caspases by the inhibitor of apoptosis protein (IAP) family can downregulate the apoptotic response. In some systems, IAP self-ubiquitination and degradation is required for full apoptosis activation [4], while in others free Smac/DIABLO released from the mitochondria can bind IAPs and inhibit their anti-apoptotic activity [5]. The ability of Smac mimetic compounds that target IAP activity to sensitize cancer cells to chemotherapeutic agents is currently being tested in clinical trials [6].

There are two major apoptotic-inducing pathways, intrinsic (mitochondrial) and extrinsic (death receptor-mediated). The involvement of ubiquitination in the regulation of apoptosis has largely been assessed in the intrinsic apoptotic pathway (i.e., Mdm2/p53, Smac/IAP). In contrast, much less is known regarding the ubiquitination machinery or ubiquitin substrates that regulate cell-extrinsic apoptosis. Death receptor-mediated apoptosis is induced by the binding of death ligands to their corresponding death receptors at the plasma membrane [7]. Death ligands are members of the tumor necrosis factor (TNF) family of ligands such as Fas Ligand (FasL) and TNF related apoptosis inducing ligand (TRAIL). Clustering of death receptors upon ligation allows for the formation of the death inducing signaling complex (DISC) [8]. Procaspase-8 present at the DISC, and possibly in other high molecular weight aggregates, is autocatalytically processed to form an active p10/p18 homodimer [9]. Active caspase-8 can cleave effector caspases-3 and -7, as well as other death substrates, fully inducing the downstream apoptotic pathway.

Here, using a high throughput siRNA screen we identified two E3 ubiquitin ligases, Siah2 (Seven in absentia homologue) and SH3RF1 (SH3 domain containing RING finger 1, also known as POSH for plenty of SH3 domains), as negative regulators of death receptor mediated apoptosis through the modulation of caspase-8 activity. We also defined a physical interaction between these two E3s suggesting Siah2 and POSH may function through shared or similar signaling pathways to regulate extrinsic apoptosis.

## Results and Discussion

### A ubiquitin ligase screen identifies Siah2 and POSH as regulators of caspase-8 activity

**Figure 1:**
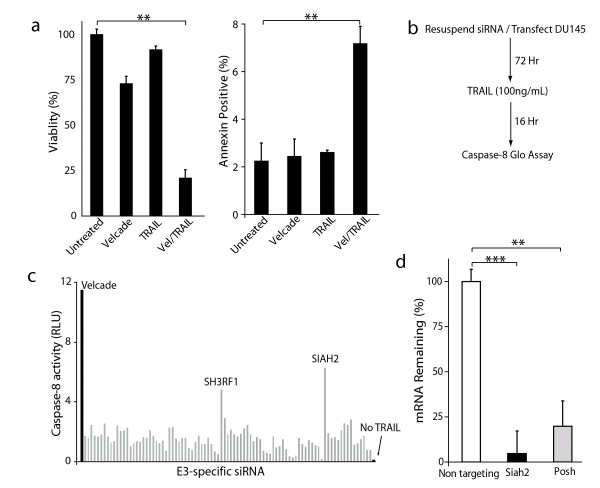
**E3 ubiquitin ligases Siah2 and POSH can regulate caspase-8 activity**. **a**: (left) MTT viability and (right) annexin V apoptosis analysis of DU145 cells treated with Velcade (50 nM), GST-TRAIL (100 ng/mL) or the combination for 24 and 16 hours, respectively. **b**: Flow chart illustrating the experimental design of the E3 ubiquitin ligase screen utilized in this study. **c**: Representative screening results showing 90 out of 239 E3 ligases examined in the siRNA screen. DU145 cells were plated/reverse-transfected for 72 hours and then treated with TRAIL (50 ng/ml) for 16 hours. Caspase-8 activity was quantified using a luminescent reporter. Values were normalized by subtracting out the background levels of caspase-8. The black bar indicates treatment with TRAIL and Velcade. Gray bars denote each TRAIL treated and siRNA targeted E3. 'No TRAIL' indicates no treatment. **d**: Assessment of Siah2 and POSH silencing in DU145 cells using qRT-PCR 24 hours post-transfection. All data are represented as the mean +/- the standard deviation. *P < 0.05, **P < 0.01, ***P < 0.001 using a two-tailed Student's *t*-test.

We previously demonstrated the ability of proteasome inhibition to sensitize cancer cells to death receptor-mediated apoptosis both in vitro and in vivo [10,11]. The goal of this study was to identify ubiquitin ligases that could impair death receptor signaling. DU145 prostate cancer cells were selected as a model to address this question because of their low levels of background caspase-8 activity. First, to confirm the synergy between proteasome inhibition and death ligand signaling in the DU145 line, cells were treated with Velcade (50 nM) and TRAIL (100 ng/mL) alone or in combination. Cells treated with the combined Velcade and TRAIL regimen displayed a significant reduction in cell viability and a corresponding increase in apoptosis as determined by annexin V staining (Figure 1a). Next, to identify possible UPS associated proteins that may be important for death receptor signaling, an E3 ligase siRNA screen was performed as outlined (Figure 1b). DU145 cells were concurrently seeded in 96-well plates and reverse-transfected with siRNA from a E3 ligase library. Following a 72-hour incubation, cells were treated with TRAIL (100 ng/mL) for 16 hours. Cells treated with the combination of Velcade and TRAIL or left untreated served as positive and negative controls, respectively. Caspase-8 activity was measured by the addition of a synthetic pro-luminogenic caspase-8 specific substrate. Results from our screen demonstrated that targeting of two E3 ligases, Siah2 and POSH, lead to an significant (*P *< 0.01) induction of caspase-8 activity in the presence of TRAIL (Figure 1c).

The ability of the siRNA pool used in the screen to reduce Siah2 or POSH mRNA levels was assessed in DU145 cells. An average knockdown of 95 and 80% was observed for Siah2 and POSH siRNA pools, respectively (Figure 1d).

### Silencing Siah2 or POSH enhances initiator and effector caspase activity in response to TRAIL

**Figure 2:**
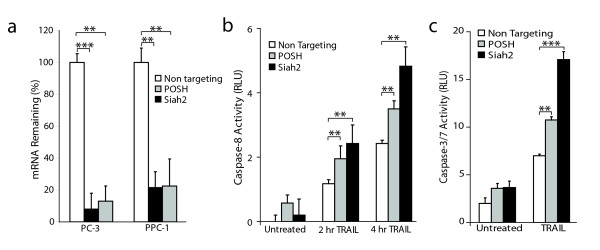
**Silencing of Siah2 or POSH enhances caspase activity in response to TRAIL**. **a**: qRT-PCR assessment of Siah2 and POSH targeting in PC-3 and PPC-1 cells 24 hours after siRNA transfection. **b**: Caspase-8 activity in PC-3 cells transfected with siRNA specific for Siah2 or POSH for 72 hours and treated with TRAIL (100 ng/mL) for 2 and 4 hours. **c**: Caspase-3/7 activity in PC-3 cells treated as in (B) for 24 hours. Statistical comparison was made using a two-tailed Student's *t*-test. **P < 0.01, ***P < 0.001.

Although the DU145 cells used above exhibit enhanced caspase-8 activity in the presence of TRAIL, they are resistant to cell death. Therefore, PC-3 and PPC-1 prostate epithelial cancer cell models were chosen for subsequent analyses based on their partial sensitivity to TRAIL-induced apoptosis [10]. First, Siah2 and POSH gene knockdown by siRNA in PC-3 and PPC-1 cells was determined by qRT-PCR. Over 90% knockdown was observed in PC-3 cells and 80% silencing in PPC-1 cells for each gene (Figure 2a).

To determine the biological consequence of Siah2/POSH silencing on caspase activity, PC-3 cells were transfected with siRNA specific for these two E3 ligases or a non-targeting control. After 72 hours, transfected cells were left untreated or treated with TRAIL (100 ng/mL) for two or four hours to obtain early values for activity of the extrinsic pathway initiator caspase, caspase-8. TRAIL treatment induced caspase-8 activity in all samples. Both Siah2 and POSH repression led to elevated levels of caspase-8 activity over the non-targeting control as early as two hours following TRAIL treatment (Figure 2b). Activity of the executioner caspases 3 and 7 were also assessed after 24 hours of TRAIL treatment. Consistent with the caspase-8 findings, silencing Siah2 or POSH led to an increase in caspase-3/7 activity (Figure 2c). These results confirm our screening data and reveal Siah2 and POSH as two E3 ubiquitin-ligase enzymes that can regulate death receptor-induced caspase activity.

### Increased sensitivity to apoptosis induced by death ligands following Siah2 or POSH silencing

**Figure 3:**
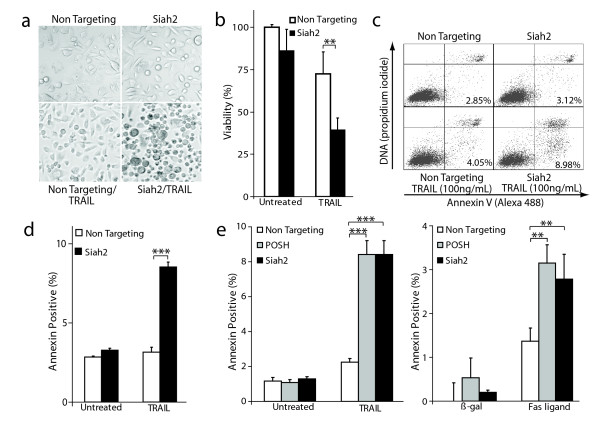
**Increased apoptosis sensitivity to death ligands by silencing Siah2/POSH**. **a**: Phase contrast micrographs of PPC-1 cells transfected with siRNA specific for Siah2 or a non-targeting control and treated +/- TRAIL (100 ng/mL) for 10 hours. **b**: Quantification of cell viability in PPC-1 cells treated as described (A) for 24 hours using an MTT assay. **c**: Apoptosis analysis. PPC-1 cells were transfected with siRNA against Siah2 or non-targeting control for 72 hours. Cells were left untreated or treated with 100 ng/mL TRAIL for 16 hours and subjected to annexin V staining. Cells in lower left quadrant are viable, annexin V negative; lower right, viable annexin V positive; upper right, dead annexin V positive. The percentage of viable, annexin V positive cells is depicted in lower right quadrant. **d**: Quantification of viable annexin V positive cells in three independent experiments. **e**: Siah2 or POSH was silenced in PC-3 cells and either treated with TRAIL for 16 hours (right) or transfected with Fas ligand or a LacZ (control) expression construct for 24 hours (left). Annexin V staining was performed and the percentage of viable, annexin V positive cells quantified. All statistical analysis was carried out using a Student's *t*-test. **P < 0.01, ***P < 0.001.

To test the effect of silencing Siah2 on the susceptibility to extrinsic apoptosis, PPC-1 cells were first transfected with Siah2-specific siRNA or a non-targeting control for 72 hours. Next, cells were treated with or without TRAIL and visualized by phase-contrast microscopy. After 10 hours, Siah2 targeting led to prominent apoptotic morphological characteristics (i.e. membrane blebbing, cell shrinkage and loss of adherence) in response to TRAIL (Figure 3a). To quantify viability, a colorimetric MTT assay was performed on the same set of cells 24 hours after TRAIL treatment. Cells in which Siah2 was targeted were significantly more susceptible to TRAIL-induced cell death than non-targeting siRNA control cells as shown by the decrease in cell viability by approximately 50% (Figure 3b).

To test if the observed differences in cell viability were due to the induction of apoptosis, annexin V staining was carried out. PPC-1 cells were transfected with Siah2 or non-targeting siRNA. Following a 72-hour incubation, TRAIL was added to the media for 16 hours. Cells were then stained with annexin V and propidium iodide and subjected to flow cytometry to identify the viable, annexin V positive cell population (Figure 3c). Siah2 silencing led to a marked induction of annexin positive cells compared to the non-targeting control (Figure 3d). The ability of Siah2 knockdown to enhance TRAIL-induced apoptosis was confirmed using a unique siRNA that targeted the 3'-UTR (Additional File 1). Consistent with the observation that targeting Siah2 increases caspase-8 activity, these findings demonstrate that Siah2 can regulate TRAIL-mediated apoptosis.

To further assess the impact of silencing Siah2 or POSH on both TRAIL and Fas ligand-mediated apoptosis the PC-3 cell line was used. After siRNA-mediated knockdown of Siah2 or POSH, either TRAIL was added or cells were transfected with a Fas ligand (FasL) expressing construct. Following 16-hour incubation with TRAIL or 24 hours post-transfection, annexin V staining was performed. In PC-3 cells containing diminished Siah2 or POSH levels, stimulation with TRAIL or FasL lead to a 4-fold and 2-fold increase in apoptosis, respectively, as compared to non-targeting siRNA control cells (Figure 3e). These data identify Siah2 and POSH as negative regulators of death receptor-mediated apoptosis.

### RING-domain function is required for the anti-apoptotic activity of Siah2 but not POSH

**Figure 4:**
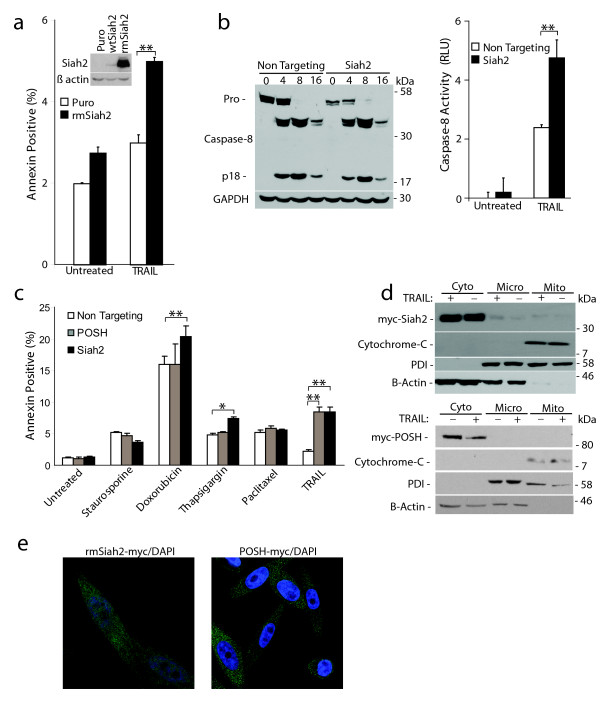
**Siah2 functions down stream of procaspase-8 processing**. **a**: Siah2 E3 activity is required to maintain apoptosis resistance. PPC-1 cells stably expressing wtSiah2, rmSiah2 or empty vector (inset) were treated with TRAIL (100 ng/mL) for 16 hours and stained for annexin V. **b**: Siah2 acts downstream of caspase-8 processing. (left) Caspase-8 Western blot on PPC-1 cell extracts transfected with Siah2 or non-targeting siRNA and TRAIL treated from 0 to 16 hours. GAPDH serves as loading control. (right) Caspase-8 activity determined on the 16 hr timepoints used in (B). **c**: PC-3 cells were transfected with siRNA to target Siah2, POSH, or a non-targeting control. After 48 hours cells were plated to a 24-well dish. After 72 hours, cells were treated with staurosporine (100 nM for 6 hours), doxorubicin (1 μM for 30 hours), thapsigargin (1 μM for 24 hours) or TRAIL (100 ng/mL for 16 hours) and annexin V stained to assess apoptosis. All values indicate the mean percent annexin V positive cells +/- the standard deviation. **d**: Western blot analysis of sub-cellular fractionation from PPC-1 cells stably expressing rmSiah2-myc (top) or rmPOSH-myc (bottom) and treated +/- TRAIL (100 ng/mL). Mito, mitochondria; cyto, cytoplasm; micro, microsomal; PDI, protein disulfide isomerase. **e**: Representative confocal images of PPC-1 cells stably expressing rmSiah2. Siah2 and proteasome localization was visualized by incubation with anti-myc and anti-20S antibodies. *P < 0.05, **P < 0.01.

Targeting Siah2 and POSH enhanced sensitivity to apoptosis. This could be due to either downregulation of protein expression or loss of enzymatic activity. To discern between these two possibilities we generated RING mutant versions of Siah2 (rmSiah2) and POSH (rmPOSH). RING-finger domains bind to ubiquitin-conjugating enzymes. This association brings a substrate in close proximity to the ubiquitin-conjugated E2. RING domains feature a distinct cross-brace motif in which conserved cysteine and histidine residues coordinate two zinc atoms [12]. Mutating a cysteine and histidine within the cross-brace abrogates E3 function by preventing association with an ubiquitin-charged E2. For Siah2, we used site-directed mutagenesis to generate H98A/C101A mutations and stably expressed the rmSiah2 construct (Figure 4a, inset). PC-3 cells stably expressing rmSiah2 or the vector only were treated with TRAIL (100 ng/mL) for 16 hours and analyzed for apoptosis induction. Inhibiting Siah2 RING function led to an increased sensitivity to TRAIL-mediated apoptosis as compared to empty vector control cells (Puro) (Figure 4a). In contrast, rmPOSH had no effect on TRAIL-induced cell death (Additional File 2). These data suggest that the enzymatic function of Siah2 and the protein levels of POSH are responsible for their ability to regulate apoptosis.

### Siah2 functions downstream of caspase-8 processing

To begin to identify where in the apoptotic pathway Siah2 functions, the effect of Siah2 expression on procaspase-8 processing was examined. PPC-1 cells were transfected with siRNA to silence Siah2 or a non-targeting control sequence. After 72 hours, cells were treated with TRAIL and procaspase-8 processing was monitored by Western blot analysis. Cells transfected with non-targeting siRNA and treated with TRAIL showed a decrease in procaspase-8 abundance and a corresponding increase in the catalytically active p18 subunit. p18 subunit abundance was highest eight hours after treatment and decreased over 16 hours. Similar procaspase-8 processing kinetics was observed in Siah2 knockdown cells (Figure 4b, left). However, silencing of Siah2 in PPC-1 cells leads to an increase in caspase-8 activity upon TRAIL stimulation (Figure 4b, right). These data imply that Siah2 does not function to regulate procaspase-8 processing, but rather mediates its effects further downstream in the apoptotic pathway.

As Siah2 silencing was enhancing caspase-8 activity downstream of DISC processing we hypothesized that this E3 may also affect cell death outside of the extrinsic pathway. Therefore, its ability to impact cell death induced by other agents was investigated. PC-3 cells in which Siah2 was targeted were treated with various agents that induce apoptosis through multiple pathways and the effect on apoptosis was quantified. Although not as robust as when exposed to TRAIL, Siah2 silencing resulted in sensitization to doxorubicin and thapsigargin (Figure 4c). In contrast, POSH silencing only sensitized cells to extrinsic death stimuli (Figure 4c). These data demonstrate that agents that function independent of the extrinsic pathway also achieve apoptosis induction following Siah2 silencing.

Sub-cellular localization was performed to gain further insight into where Siah2 and POSH may function. Protein from PPC-1 cells stably overexpressing rmSiah2-myc or POSH-myc was harvested and subjected to fractionation. Siah2 and POSH were present only in the cytoplasmic fractions and their presence was unaltered upon TRAIL stimulation (Figure 4d). Cytosolic localization was confirmed by immunocytochemistry and confocal microscopy. Confocal analysis revealed Siah2 to be primarily cytoplasmic with an intense punctuate, granular staining pattern reminiscent of the 26S proteasome (Figure 4e). Subsequent immunocytochemistry performed in conjunction with anti-20S proteasome antisera revealed high levels of co-localization (Figure 4e). As these localization experiments utilize the overexpression of a tagged construct, it is possible that endogenous Siah2 is not localized in the same exact manner. However, these data suggest Siah2 is acting to inhibit cell death downstream in the apoptotic pathway, possibly mediating its function at the proteasome.

### Siah2/POSH interaction

**Figure 5:**
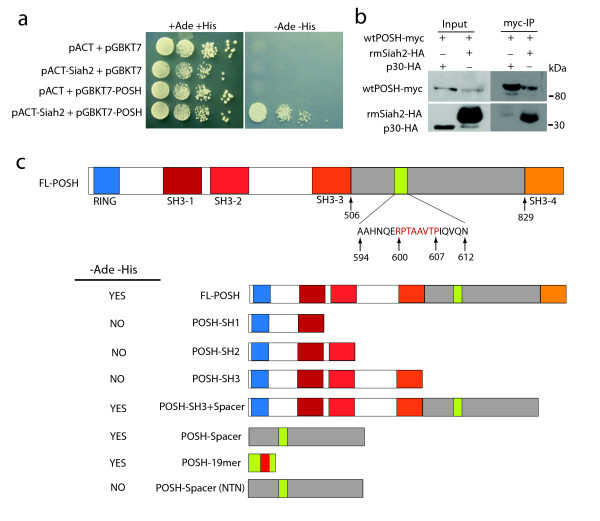
**Physical interaction between POSH and Siah2**. **a**: The AH-109 yeast strain was co-transformed with full length POSH Gal4-DNA-binding-domain fusion construct (pGBKT7-POSH) and wild type Siah2 GAL4-activation-domain fusion construct (pACT-Siah2) or empty vector controls. Transformants were grown on plates supplemented with (+Ade+His) or without (-Ade-His) adenine and histidine. -Ade-His is the yeast-medium-plate lacking Leu, Trp, His and Ade in which activation of the adenine and histidine reporter gene is being tested. +Ade+His is the yeast-medium-plate lacking Leu and Trp, selecting only for presence of plasmids being tested for interaction. **b**: PPC-1 cells stably expressing POSH-myc were transfected with rmSiah2-HA or caspase-8 p30 as a negative control and co-immunoprecipitation using anti-myc conjugated magnetic beads was performed. The anti-myc immune complexes were analyzed for the presence of Siah2-myc via Western blot. **c**: Schematic representation of full length POSH (top) and various mutants cloned into the pGBKT7 vector. The yeast strain AH-109 was co-transformed with pACT-Siah2 and the various POSH mutants and grown on +Ade+His or -Ade-His plates. YES/NO indicates the presence or absence of colonies on -Ade-His plates (left).

Surprisingly, it has been demonstrated that POSH, in an E3-independent fashion, interacts with Siah2 and can stabilize Siah proteins [13]. To verify this interaction, a yeast two-hybrid assay was employed. The AH-109 yeast strain was co-transformed with full length POSH Gal4-DNA binding-domain fusion construct (pGBKT7-POSH) and wild type Siah2 GAL4-activation-domain fusion construct (pACT-Siah2) or empty vector controls. Although all co-transformants grew on +Ade+His plates, only those co-transformed with constructs expressing both POSH and Siah2 grew on the -Ade-His indicating an interaction has occurred (Figure 5a). To verify an interaction between Siah2 and POSH PPC-1 cells stably expressing wtPOSH-myc were transiently transfected with rmSiah2-HA or caspase-8 p30-HA as a negative control. Co-immunoprecipitation using anti-myc conjugated magnetic beads revealed rmSiah2 present in the wtPOSH-myc precipitate but not p30-HA (Figure 5b)

To determine where Siah2 was binding to POSH, several POSH deletion mutants containing individual or combinations of SH3-domains were generated (Figure 5c) and a yeast two-hybrid mapping analysis was performed. As shown in Figure 5c, only the yeast transformants containing the POSH spacer region between SH3-3 and SH3-4 could bind to Siah2 and induce reporter gene transcription. In fact, the spacer region of POSH alone was sufficient to induce POSH/Siah2 binding. Interestingly, a known Siah2 substrate binding motif (RPxAxVxP) is present within the spacer region and could be mediating POSH binding to Siah2. Yeast transformants expressing wtSiah2 in combination with a 19AA portion of the POSH spacer region containing the Siah2 binding motif (POSH-19mer) also grew on -Ade-His suggesting POSH binds the C-terminal substrate binding domain of Siah2. To further validate this finding, a POSH construct was generated in which two key residues within the Siah2 binding domain were mutated (POSH-spacer-NTN). Mutating the suspected Siah2 binding domain within the POSH spacer region abolished POSH/Siah2 interaction (Figure 5c) and confirmed POSH binding to the substrate binding domain of Siah2.

## Conclusion

The primary finding presented here is the ability of two E3 ubiquitin ligases to weaken signaling through the death receptor apoptotic pathway. By utilizing RNA interference technologies we have successfully established Siah2 and POSH as two UPS components that can reduce caspase activity in response to death ligands in prostate cancer cells.

Siah2 is a homolog of the *Drosophila *Sina gene, a critical component in photoreceptor development. The protein contains an N-terminal RING-finger domain which provides E3 ubiquitin ligase activity. The RING-domain is followed by two zinc finger-domains and a C-terminal substrate binding-domain [14]. Current literature provides evidence for Siah2 function in the hypoxic response. One proposed mechanism is by regulating HIF-1α protein levels by the ubiquitination and subsequent degradation of PHD3 (prolyl hydroxylating domain-containing 3), a prolyl hydroxylase that controls HIF-1α accumulation in hypoxic environments [15]. A second possible hypoxia regulatory point is through the degradation of HIP2K (homeodomain-interacting protein kinase 2), a member of the HIPK family of proline-directed kinases [16]. Under normoxia, the ability of Siah2 to modulate cell proliferation in lung cancer cells has been detailed [17]; a finding we have confirmed in prostate cancer cells (Additional File 3).

POSH (plenty of SH3s) is a large scaffold molecule containing an N-terminal RING-finger domain followed by four SH3-domains and a region known to bind Rac1-GTP. The RING-domain has been shown to display ubiquitin ligase activity and is thought to regulate trans-Golgi network transport [18]. The scaffolding function of POSH is most closely associated with c-Jun N-terminal kinase signaling [19]. Although multiple investigators have reported transient POSH overexpression can induce apoptosis [19,20], we did not observe death in our prostate cancer models upon lower, stable expression (data not shown). Conversely, we have shown that down regulation of POSH can sensitize cells to TRAIL-dependent apoptosis induction.

Providing mechanistic data to explain Siah2-dependent apoptosis regulation is ongoing. Initial Siah2 characterizations in the death receptor pathway have revealed clues to possible mechanisms. As shown in Figure 4a, the E3 activity of Siah2's RING-domain is required for its anti-apoptotic function, suggesting the E3 ligase enzymatic activity is responsible for the degradation of a pro-apoptotic protein. Proposed Siah2 substrates include TRAF2, PHD3 and HIPK2 [15,16,21]. We have failed to confirm the interaction with PHD3 or TRAF2 or Siah2-mediated regulation of NF-κB activity in our prostate models (data not shown). However, the recently identified pro-apoptotic, Siah2-specific substrate HIPK2 is a promising candidate. HIPK2 is a serine/threonine kinase known to phosphorylate p53 at serine 46, thereby promoting p53 acetylation and p53-dependent gene expression [22]. Inhibition of HIPK2 has been shown to protect lung cancer cells against UV-induced apoptosis while overexpression of HIPK2 sensitizes cells to UV-induced apoptosis and decreases cell proliferation [22,23]. Together, these data suggest a possible scenario in which Siah2 targeting of HIPK2 protects cancer cells from apoptosis induction while inhibition of Siah2 could increase HIPK2 abundance thus sensitizing cancer cells to cell death agents.

Regulation of cell death by Siah2 was found to be selective, but not specific for the death receptor pathway. This finding, along with data demonstrating Siah2 silencing induces caspase-8 activity without altering procaspase-8 processing, suggested to us that Siah2 was acting further downstream in the death receptor pathway. Cellular localization analysis of Siah2 revealed a predominately cytoplasmic staining pattern, consistent with previous reports [16,24]. High levels of Siah2 co-localization with the 20S core proteasome was also observed and may provide insight into Siah2's regulation of cell death. Interestingly, several E3-ligases such as Hul5, Parkin, Ubr1, and VHL are known to localize to complexes in association with the 26S proteasome [25], adding validity to this novel finding.

In contrast to Siah2, the ability of POSH to regulate apoptosis induction in response to TRAIL was independent of E3 activity, suggesting protein-protein interactions (possibly mediated through one of four SH3-domains) are important for this activity. Surprisingly, it has been demonstrated that POSH in an E3-independent fashion interacts with Siah2 and can stabilize Siah proteins [13]. We have successfully confirmed a physical interaction between E3 ligases Siah2 and POSH. By utilizing a yeast-two-hybrid mapping approach we have defined the spacer region of POSH, more specifically the RPxAxVxP Siah2 substrate binding motif encompassing amino acids 601-607, to be the site of Siah2 binding. POSH has been shown to bind both Siah1 and Siah2 suggestive of possible overlapping, POSH-dependent functions of these two E3s. The RPxAxVxP motif within POSH is highly conserved from *Xenopus *to humans suggesting a Siah-POSH binding event is critical for the proper function of a highly conserved biochemical pathway. Taken together, the ability of Siah2 to interact with such a large signaling molecule such as POSH, as well as its capacity to dimerize, could provide opportunities for Siah2 to be recruited to key protein signaling complexes where it can carry out its E3 ligase function. For example, monomeric Siah2 could be recruited to signaling complexes via a degradation-independent interaction with POSH. Free monomeric Siah2 could then associate with the complex through dimerization. POSH, through one of four SH3 domains, could recruit possible target substrates allowing Siah2 to carry out its polyubiquitination/degradation function.

As Siah2 and POSH suppress cell death, their upregulation may be advantageous in cancer cells. We, therefore, speculated that these E3s would be overexpressed in a subset of cancers. Oncomine analysis revealed Siah2 to be significantly overexpressed in both B-cell acute lymphoblastic leukemia [26] and squamous non-small-cell lung carcinoma [27], while POSH is one of the most frequently overexpressed genes in prostate cancer [28-30]. At this point, however, there is no evidence that either gene is capable of cellular transformation.

Together, these data suggest that development of second generation proteasome inhibitors that block E3 ubiquitin ligases, such as Siah2 and POSH, could be beneficial for cancer treatment. Recently, major advances in targeted E3 therapies for the intrinsic apoptotic pathway have been made. Most notably, generation and characterization of Smac mimetic compounds that inhibit IAP function. Smac mimetic compounds have been shown to sensitize cancer cells to chemotherapeutic agents as wells as strongly enhance the anti-tumor activity of death ligands such as TRAIL in vivo [6]. It remains to be tested if targeting of the extrinsic apoptotic pathway via Siah2 or POSH inhibition in combination with current intrinsic targeting compounds is a promising cancer therapeutic strategy.

## Methods

### Cell Culture and Reagents

All prostate cancer cell lines were obtained from ATCC (Manassas, VA) and grown in DMEM containing 10% FBS. Velcade (Millenium Pharmaceuticals, Cambridge, MA) was donated by the University of Kentucky, Markey Cancer Center Pharmacy. The GST-TRAIL fusion protein was purified by affinity chromatography as described [31]. Fas ligand was expressed in cells as described [10]. Staurosporine, doxorubicin and thapsigargin were purchased from EMD Biosciences (San Diego, CA).

### Cell Viability, Apoptosis and Proliferation Assays

Cell viability was measured using a colorimetric MTT assay. Media was removed from the cells and a 1 mg/ml solution of thiazolyl blue tetrazolium bromide (MTT) (Alfa Aesar, Ward Hill, MA) in 1 × PBS was added. The samples were read on a spectrophotometer at 570 nm minus 690 nm. All assays were conducted in replicates of four to six. Annexin V staining was carried out as described by the manufacturer (Invitrogen, Carlsbad, Ca). Cells were immediately analyzed by flow cytometry (FACSCalibur, BD Immunocytometry Systems, San Jose, CA). CellQuest Pro software (BD Immunocytometry Systems) was used to quantify percentage of annexin V positive and viable cells in 20,000 gated events.

BrdU labeling and flow cytometric analysis was carried out as previously described [32]. The percentage of BrdU positive cells (10,000 gated events) was determined using CellQuest software (BD). All assays were carried out in replicates of three. All statistical assessments were made using a Student's t-test.

### Western Blot Analysis

Cell lysates were prepared in ubiquitin protein extraction buffer (UPEB; 150 mM NaCl, 50 mM Tris-HCl (pH 7.5), 5 mM EDTA, 1% (v/v) NP-40, 0.1% (w/v) SDS, 0.5% (w/v) sodium deoxycholate). Western blot analysis was carried out as previously described [33]. Antibodies were obtained from the following sources: β-actin (Sigma, St. Louis, MO), caspase-8, 1C12 (Cell Signaling Technology, Danvers, MA), myc, 9B11 (Cell Signaling Technology), cytochrome-C (Santa Cruz Biotechnology, Santa Cruz, CA), PDI (Stressgene, Ann Arbor, MI).

### Caspase Activity

In vivo caspase-8 activity was measured with the luminescent-based Caspase-Glo 8 Assay kit while caspase-3/7 activity was measured by the addition of the luminescent-based Caspase-Glo 3/7 Assay kit (Promega, Madison, WI). At 16 hr post-treatment, three wells in each treatment group were pre-incubated for 30 min with the cell permeable caspase-8 inhibitor Z-IETD-fmk or the pan caspase inhibitor Z-VAD fmk (25 mM, R&D Systems, Minneapolis, MN). Each treatment group consisted of seven wells, four without fmk inhibitor and three with the inhibitor. The Caspase-Glo reagent was added to the cells and activity measured with a Lmax luminometer (Molecular Devices Corporation, Sunnyvale, CA). Data was analyzed by SOFTmax Pro (Version 1.1L). Specific caspase activity was calculated by subtracting the activity derived from cells treated with Z-IETD/VAD-fmk.

### E3-ligase siRNA Screen

The siARRAY E3 ubiquitin-ligase library containing 239 total E3 specific siRNAs was purchased from Dharmacon (Lafayette, CO). The predispensed dehydrated siRNA was first rehydrated with a solution of DMEM containing DharmaFECT transfection reagent. DU145 cells (10,000) were seeded directly into the 96-well plates containing the rehydrated siRNA. 48 hours after siRNA transfection, cells were treated with GST-TRAIL (50 ng/mL) for 16 hours. The addition of 50 mM Velcade in combination with TRAIL served as a positive control. Caspase-8 activity was measured as previously discussed by the addition of Caspase-GLO 8 pro-luminescent reagent. All E3 ligase siRNA experiments were performed in triplicate.

### Quantitative Reverse-transcriptase PCR

DU145, PC-3 and PPC-1 cells were seeded into 6-well tissue culture plates. Cells were transfected with non-targeting siRNA or siRNA specific for Siah2 or POSH. 48 hours after siRNA transfection, RNA was harvested using the RNeasy kit (Qiagen, Valencia, CA). cDNA was generated using SuperScript II Reverse Transcriptase (Invitrogen). qRT-PCR was carried out using primer sets for 18S, Siah2 or POSH in the presence of FastStart Universal SYBR Green Master Mix (Roche, Nutley, NJ). PCR was performed and analyzed using a 4300 Real Time PCR System (Applied Biosystems, Foster City, CA). Percent mRNA remaining was determined by the comparative C_T _method (ΔC_T_) for relative quantification.

### E3 Ubiquitin-Ligase Cloning and Expression

Wild-type human Siah2 (NM_0056067) and RING mutant Siah2 (H98A, C101A) were generated by PCR, cloned into a retroviral pBabe puro expression vector (Addgene, Cambridge, MA) and verified by sequence analysis. Retrovirus was generated as previously described [34] and added to PC-3 and PPC-1 cells for 24 hours. Cells were selected by treatment with 2 μg/mL of puromycin (EMD Biosciences, San Diego, CA). Selection was carried out for one week and expression was confirmed by Western blotting. Similarly, wild-type POSH (NM_020870) and RING mutant POSH (H28A, C30A) were generated.

### Sub-cellular Fractionation

Cytoplasmic, microsomal and mitochondrial fractions were obtained from PPC-1 cells stably overexpressing rmSiah2-myc using the Qproteome Mitochondria Isolation Kit (Qiagen). Cells were first treated +/- GST-TRAIL (100 ng/mL) for 2 hours. Cells were collected and protein harvested in provided lysis buffer. The cytoplasmic fraction was obtained by centrifuging total cell lysate at 1000 × g for 10 min at 4°C and the collecting supernatant. Nuclei and unbroken cells were collected by pelleting lysate in the disruption buffer at 1000 × g for 10 min at 4°C. A microsomal fraction was obtained by centrifugation of protein lysate (in disruption buffer) at 6000 × g for 10 min at 4°C. Finally, the mitochondrial fraction was collected in provided mitochondrial purification buffer by sequential centrifugation at 14,000 × g for 15 min then 8000 × g for 10 min both at 4°C. The purified mitochondrial pellet was resuspended in the provided mitochondrial storage buffer. 50 μg of each fraction was added to SDS loading buffer and Western blot analysis was performed.

### Immunocytochemistry

PPC-1 cells stably expressing rmSiah2-myc were seeded into 4-well chamber slides. Cells were fixed with 4% paraformaldehyde for 20 minutes and permeabilized with saponin (1 mg/mL) in Hank's Buffered Saline Solution for 15 minutes. Following blocking in 2% BSA for 30 minutes, primary antibodies were added for 1 hour followed by PBST washing. Fluorescently conjugated secondary antibodies were added for 45 minutes. Slides were mounted in Vectashield + DAPI (Vector Laboratories, Burlingame, CA) and visualized by a Leica TSP SP5 confocal microscope. Antibodies were obtained from the following sources: myc, 9B11 (Cell Signaling Technology), 20S proteasome core subunits (Calbiochem, San Diego, CA).

### Myc-POSH Immunoprecipitation

Cells expressing myc-tagged constructs were incubated at 37°C for 48 hrs. Protein was harvested in IP buffer (10 mM Tris pH 7.4, 1% Triton X-100, 0.5% NP-40, 150 mM NaCl, 20 mM NaF, 1 mM EDTA) and incubated on ice for 30 min. Cell debris was pelleted by centrifugation at 10,000 × g for 15 min at 4°C. 50 μl μMACS anti-myc MicroBeads (Miltenyi Biotech, Auburn, CA) was added to the lysate and incubated on ice for 30 min. myc-POSH was purified by passing cell lysate + microbeads through a μMACS column attached to the μMACS magnetic Separator. Column was washed with 800 μl IP buffer and protein was eluted by adding 20 μl pre-heated (95°C) elution buffer (provided), incubating for 5 min and adding another 50 μl elution buffer. Eluted protein was subjected to SDS-PAGE followed by Western blot analysis.

### Yeast Two-Hybrid

Full length POSH was cloned into pGBKT7 (Clontech, Mountain View, CA) as a Gal4-DNA-binding domain fusion. Full length Siah2 as cloned into pACT2 (Clontech) as a Gal4-Activiation domain fusion. The fusion constructs or empty vector controls were co-transformed into the AH-109 yeast strain. Briefly, a single colony of AH-109 yeast (Clontech) was inoculated into 5 mL liquid YPD and incubated overnight at 30°C. The overnight culture was inoculated into 50 mL liquid YPD and allowed to grow to a cell density of 2 × 10^7 ^cell/mL. The 50 mL culture was harvested by centrifugation at 3000 × g for 5 min. Yeast were resuspended in 1 ml of 100 mM LiAc. Cells were pelleted at 10,000 rpm in a table top centrifuge for 5 sec and the LiAc was removed. Yeast cells were resuspended to a final cell density of 2 × 10^9 ^cells/mL in 100 mM LiAc. 50 μl of resuspended yeast were used per co-transformation. A transformation mix containing 240 μl PEG (Bainbridge Island, WA) (50% w/v), 36 μl 1.0 M LiAc, 25 μl single stranded DNA (2.0 mg/mL), plasmid DNA (2 μg per construct) and ddH_2_O up to 350 μl was made. The transformation mix was added directly to the resuspended yeast, vortexed and incubated at 30°C for 30 min. Yeast were heat shocked at 42°C for 25 min and microcentrifuged at 8,000 rpm for 15 sec. Transformation mix was removed and yeast were resuspended in 400 μl ddH_2_O. Transformed yeast were plated onto selective agar plates (200 μl per plate).

## Competing interests

The authors declare that they have no competing interests.

## Authors' contributions

PC carried out the E3 screen and subsequent Siah2 and POSH characterization and drafted the manuscript. MF carried out the JNK phosphorylation studies. SS conceived of the study and participated in its design and coordination. All authors read and approved the final manuscript.

## Supplementary Material

Additional file 1 **Targeting Siah2 by a second independent siRNA enhances apoptosis in response to TRAIL**. (left) qRT-PCR confirmation of Siah2 targeting in PC-3 cells using a Siah2 3'UTR specific siRNA pool. (right) Annexin V staining of Siah2 3'UTR targeted PC-3 cells treated with TRAIL for 16 hours (100 ng/mL). **P < 0.01, ***P < 0.001. Click here for file

Additional file 2 **RING mutant POSH expression does not affect sensitivity to TRAIL-mediated apoptosis**. PPC-1 cells stably expressing rmPOSH were treated with TRAIL for 16 hours (100 ng/mL) and annexin V stained to determine percent apoptosis. All values indicate the mean percent annexin V positive cells +/- the standard deviation. Click here for file

Additional file 3 **Cellular proliferation is decreased upon Siah2 silencing**. PC-3 cells were transfected with siRNA to target Siah2. Cells were BrdU labeled 72 hours later. The percentage of BrdU positive cells was determined by flow cytometry. All values indicate the mean BrdU positive cells (N = 3) +/- the standard deviation. ***P < 0.001. Click here for file
