# Supplementary material for: Possible role of death receptor-mediated apoptosis by the E3 ubiquitin ligases Siah2 and POSH
Source: Mol Cancer. 2011 May 17;10:57. doi: 10.1186/1476-4598-10-57 (PMC3115909; doi:10.1186/1476-4598-10-57)

Supplemental Figure 1 a: Targeting Siah2 by a second independent siRNA enhances apoptosis in response to TRAIL. (left) qRT-PCR confirmation of Siah2 targeting in PC-3 cells using a Siah2 3'UTR specific siRNA. (right) Annexin V staining of Siah2 3'UTR targeted PC-3 cells treated with TRAIL for 16 hours (100 ng/mL). **P < 0.01, ***P < 0.001. **b:** Siah1 mRNA levels do not decrease upon Siah2 silencing. Siah1 mRNA was quantified via qRT-PCR 24 hours after transfection of PC-3 cells with either Siah2 siRNA pool or Siah2-3'UTR siRNA.

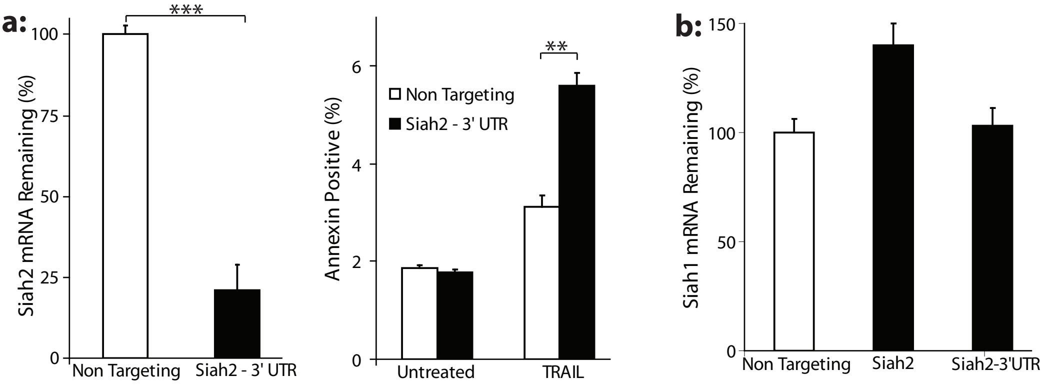

Supplement: Additional file 1 — Targeting Siah2 by a second independent siRNA enhances apoptosis in response to TRAIL. (left) qRT-PCR confirmation of Siah2 targeting in PC-3 cells using a Siah2 3'UTR specific siRNA pool. (right) Annexin V staining of Siah2 3'UTR targeted PC-3 cells treated with TRAIL for 16 hours (100 ng/mL). **P < 0.01, ***P < 0.001. [file 1476-4598-10-57-S1.PDF]
