# Supplementary material for: Possible role of death receptor-mediated apoptosis by the E3 ubiquitin ligases Siah2 and POSH
Source: Mol Cancer. 2011 May 17;10:57. doi: 10.1186/1476-4598-10-57 (PMC3115909; doi:10.1186/1476-4598-10-57)

Supplemental Figure 2 RING mutant POSH expression does not affect sensitivity to TRAIL-mediated apoptosis. PPC-1 cells stably expressing rmPOSH were treated with TRAIL for 16 hours (100 ng/mL) and annexin V stained to determine percent apoptosis. All values indicate the mean percent annexin V positive cells +/- the standard deviation.

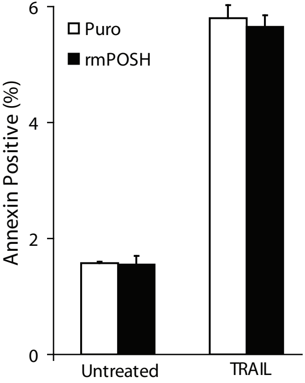

Supplement: Additional file 2 — RING mutant POSH expression does not affect sensitivity to TRAIL-mediated apoptosis. PPC-1 cells stably expressing rmPOSH were treated with TRAIL for 16 hours (100 ng/mL) and annexin V stained to determine percent apoptosis. All values indicate the mean percent annexin V positive cells +/- the standard deviation. [file 1476-4598-10-57-S2.PDF]
