# Supplementary material for: Possible role of death receptor-mediated apoptosis by the E3 ubiquitin ligases Siah2 and POSH
Source: Mol Cancer. 2011 May 17;10:57. doi: 10.1186/1476-4598-10-57 (PMC3115909; doi:10.1186/1476-4598-10-57)

Supplemental Figure 3 Cellular proliferation is decreased upon Siah2 silencing. PC-3 cells were transfected with siRNA to target Siah2. Cells were BrdU labeled 72 hours later. The percentage of BrdU positive cells was determined by flow cytometry. All values indicate the mean BrdU positive cells (N=3) +/- the standard deviation. ***P < 0.001.

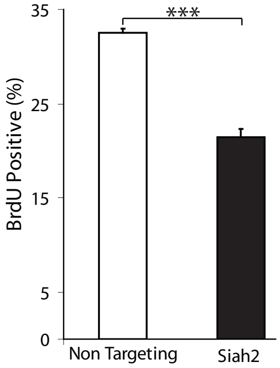

Supplement: Additional file 3 — Cellular proliferation is decreased upon Siah2 silencing. PC-3 cells were transfected with siRNA to target Siah2. Cells were BrdU labeled 72 hours later. The percentage of BrdU positive cells was determined by flow cytometry. All values indicate the mean BrdU positive cells (N = 3) +/- the standard deviation. ***P < 0.001. [file 1476-4598-10-57-S3.PDF]
